# How well do health professionals interpret diagnostic information? A systematic review

**DOI:** 10.1136/bmjopen-2015-008155

**Published:** 2015-07-28

**Authors:** Penny F Whiting, Clare Davenport, Catherine Jameson, Margaret Burke, Jonathan A C Sterne, Chris Hyde, Yoav Ben-Shlomo

**Affiliations:** 1 School of Social and Community Medicine, University of Bristol, Bristol, UK; 2 The National Institute for Health Research Collaboration for Leadership in Applied Health Research and Care West at University Hospitals Bristol NHS Foundation Trust; 3 Unit of Public Health, Epidemiology and Biostatistics, School of Health and Population Sciences, College of Medical and Dental Sciences, University of Birmingham, Edgbaston, Birmingham, UK; 4 Peninsula Technology Assessment Group, Peninsula College of Medicine & Dentistry, Exeter, UK

**Keywords:** EPIDEMIOLOGY, MEDICAL EDUCATION & TRAINING, STATISTICS & RESEARCH METHODS

## Abstract

**Objective:**

To evaluate whether clinicians differ in how they evaluate and interpret diagnostic test information.

**Design:**

Systematic review.

**Data sources:**

MEDLINE, EMBASE and PsycINFO from inception to September 2013; bibliographies of retrieved studies, experts and citation search of key included studies.

**Eligibility criteria for selecting studies:**

Primary studies that provided information on the accuracy of any diagnostic test (eg, sensitivity, specificity, likelihood ratios) to health professionals and that reported outcomes relating to their understanding of information on or implications of test accuracy.

**Results:**

We included 24 studies. 6 assessed ability to define accuracy metrics: health professionals were less likely to identify the correct definition of likelihood ratios than of sensitivity and specificity. –25 studies assessed Bayesian reasoning. Most assessed the influence of a positive test result on the probability of disease: they generally found health professionals’ estimation of post-test probability to be poor, with a tendency to overestimation. 3 studies found that approaches based on likelihood ratios resulted in more accurate estimates of post-test probability than approaches based on estimates of sensitivity and specificity alone, while 3 found less accurate estimates. 5 studies found that presenting natural frequencies rather than probabilities improved post-test probability estimation and speed of calculations.

**Conclusions:**

Commonly used measures of test accuracy are poorly understood by health professionals. Reporting test accuracy using natural frequencies and visual aids may facilitate improved understanding and better estimation of the post-test probability of disease.

**Strengths and limitations of this study**

- This is the first systematic review of health professionals’ understanding of diagnostic information.
- We conducted extensive literature searches in an attempt to maximise retrieval of relevant studies.
- We did not perform a formal risk of bias assessment as study designs included in the review varied and most were single-group studies that examined how well doctors could perform certain calculations or understand pieces of diagnostic information. There is no accepted tool for assessing the risk of bias in these types of study and so we were unable to provide a formal assessment of risk of bias in these studies.

## Introduction

Making a correct diagnosis is a prerequisite for appropriate management.^1^ Probabilistic reasoning is suggested to be a prominent feature of diagnostic decision-making,^2^ ^3^ but the extent to which this is based on quantitative revision of health professionals’ estimated pretest probabilities, rather than intuitive judgements, is not known.

Test accuracy can be summarised using a range of measures derived from a 2×2 contingency table (table 1). Measures that distinguish between the implications of a positive test result (positive predictive value (PPV), positive likelihood ratio (LR), specificity) and a negative test result (negative predictive value, negative LR, sensitivity) are more useful for decision-making than global test accuracy measures such as diagnostic ORs and the area under the curve (AUC).^4–6^ Predictive values and LRs, which are applied based on the test result, are believed to be more clinically intuitive than sensitivity and specificity, which are applied based on disease status.^7^ ^8^ The promotion of evidence-based testing, including the use of LRs,^8–10^ is based on the premise that formal probabilistic reasoning is necessary for informed diagnostic decision-making.^11^ ^12^ Such reasoning requires use of Bayes’ theorem to revise the pretest odds of disease, based on the test result, to give the post-test odds of disease.^13^

**Table 1:** A 2×2 table showing the cross-classification of index test and reference standard results and overview of measures of accuracy that can be calculated from these data*

	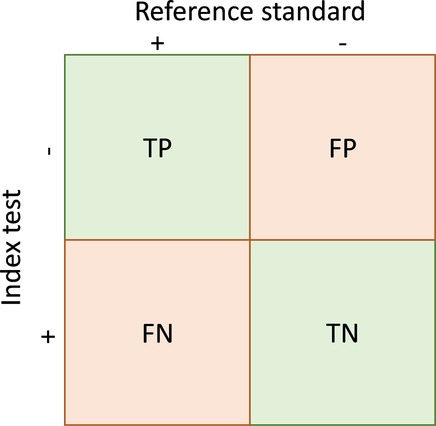	
True positives	People with the target condition who have a positive test result	TP
True negatives	People without the target condition who have a negative test result	TN
False positives	People without the target condition who have a positive test result	FP
False negatives	People with the target condition who have a negative test result	FN
Sensitivity	Proportion of patients with the target condition who have a positive test result	TP/(TP+FN)
Specificity	Proportion of patients without the target condition who have a negative test result	TN/(FP+TN)
Positive predictive value (PPV)	Probability that a patient with a positive test result has the target condition	TP/(TP+FP)
Negative predictive value (NPV)	Probability that a patient with a negative test result does not have the target condition	TN/(FN+TN)
Prevalence	The proportion of patients in the whole study population who have the target condition	(TP+FN)/(TP+FP+FN+TN)
Positive likelihood ratio (LR+)	The number of times more likely a person with the target condition is to have a positive test result compared with a person without the target condition	(TP/(TP+FN))/(FP/(FP+TN)) or sensitivity/(1−specificity)
Negative likelihood ratio (LR−)	The number of times more likely a person with the target condition is to have a negative test result compared with a person without the target condition	(FN/(TP+FN))/(TN/(FP+TN)) or (1−sensitivity)/specificity

*Adapted from Whiting P, Martin RM, Ben-Shlomo Y, et al. How to apply the results of a research paper on diagnosis to your patient. JRSM Short Reports 2013;4:7.

FN, False negatives; TP, true positives.

There is a widespread belief that health professionals and decision-makers have difficulty understanding and applying test accuracy evidence.^14^ ^15^ Difficulties are thought to arise from the need to interpret conditional probabilities, and the complex nature of probability revision. However, to date there has been no systematic review of the literature pertaining to clinician's understanding of test accuracy evidence. Here, we aimed to evaluate whether clinicians differ in how they evaluate and interpret different diagnostic test information. The findings will be used to provide recommendations about how the results of test accuracy research should be presented in order to promote evidence-based testing.

## Methods

We followed standard systematic review methods^16^ and established a protocol for the review (available from the authors on request).

### Data sources

We searched MEDLINE, EMBASE and PsycINFO from inception to September 2013. We combined terms for *measures of accuracy* AND terms for *communicating and interpreting* AND terms for *health professionals* (see web appendix 1). Additional studies were identified by screening the bibliographies of retrieved studies, contacting experts and through a citation search of four key included studies that is, identifying studies that had cited these papers.^17–20^ Contacting experts involved presenting results at a national conference and obtaining literature passively through discussions with experts at national and international conferences and meetings concerned with test evaluation. No language or publication restrictions were applied.

### Inclusion criteria

Primary studies of any design that provided information on the accuracy of any diagnostic test (eg, sensitivity, specificity, LRs, predictive values, and receiver operator characteristic (ROC) plots/curves) to health professionals (eg, doctors, nurses, physiotherapists, midwives), or student health professionals, from any specialty and that reported outcomes relating to their understanding of test accuracy were eligible for inclusion. Studies were screened for relevance independently by two reviewers; disagreements were resolved through consensus. Full-text articles of studies considered potentially relevant were assessed for inclusion by one reviewer and checked by a second.

### Data extraction

Data extraction was carried out by one reviewer and checked by a second using a standardised form. Study quality was not formally assessed due to a lack of any agreed tools for studies of this type.

### Synthesis

We combined results using a narrative synthesis due to heterogeneity between studies in terms of design, type of health professionals and measures of accuracy investigated, making a quantitative summary (meta-analysis) inappropriate. We grouped studies according to their objective: (1) accuracy definition (ability to define measures of accuracy); (2) self-reported understanding (doctors self-rating of their understanding or use of accuracy measures); (3) assess Bayesian reasoning (combining data on the pretest probability of disease with accuracy measures to obtain information on the post-test probability of disease) and (4) presentation format (impact of presenting accuracy data as frequencies rather than probabilities). Groupings were defined based on the data.

## Results

**Figure 1:**
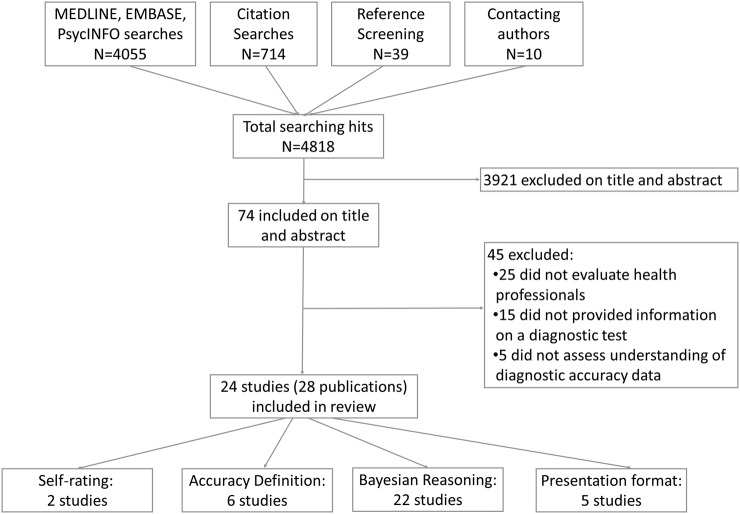
Flow of studies through the review process.

The searches identified 4808 records of which 24 studies reported in 28 publications^17^ ^19–45^ were included in the review (figure 1). Table 2 presents a summary of the included studies, grouped according to objective; further details are provided in web appendix 2. The majority of studies investigated health professionals understanding of sensitivity and specificity (or false-positive rate), six studies assessed LRs and two studies assessed other measures such as graphical displays. Only one study assessed a global measure of accuracy, the ROC curve, this was a study of doctors’ self-reported understanding. Box 1 provides examples of some of the types of scenario used in the included studies.

Box 1 **Example of population based scenarios and clinical vignettes**

*Self-rating of understanding*:^41^

QUESTIONS USED IN TELEPHONE SURVEY

1. Some authorities recommend that diagnostic decisions be made first by obtaining a test's sensitivity and specificity, estimating the prevalence of disease (in the patient under evaluation), then calculating a positive or negative predictive value. Do you perform these calculations when you make diagnostic decisions? If no, can you tell me why you do not do them?
2. Many authorities recommend that we use receiver operator characteristic (ROC) curves to set test thresholds before making diagnostic decisions. Do you use ROC curves? If no, why not?
3. Another recommendation is to use test likelihood ratios for certain diagnostic calculations. Do you use likelihood ratios before ordering tests or when interpreting test results? If no, why not?
4. Do you use test sensitivity and specificity values when you order tests or interpret test results? (For positive responses) Can you tell me in what way you use them?
5. When you use sensitivity and specificity, where do you get your values from?
6. Do you prefer to use published values for sensitivity and specificity, or values based on your clinical experience with the test?
7. Do you use positive and negative predictive accuracies when you interpret test results?
8. Do you use any other methods to help you determine the effectiveness, or accuracy of the tests you use in practice?
9. During your medical training either in medical school, residency, or perhaps fellowship training, did you participate in any formal educational activities to teach you how to use test sensitivity, specificity, or likelihood ratios?
10. Since finishing your medical training have you participated in any formal educational activities such as seminars, workshops, or CME courses designed to teach you how to use test sensitivity and specificity or likelihood ratios?

*Accuracy definition*:^40^

The sensitivity of a test is: *Please check the correct answer*

the percentage of false positive test results………………………………………..

the percentage of false negative test results………………………………………..

the percentage of persons with disease having a positive test result……………

the percentage of persons without the disease having a negative test result…

*Population based scenario: Bayesian reasoning and presentation format*^33^

Probability format

The probability that one of these women has breast cancer is 1%. If a woman has breast cancer, the probability is 80% that she will have a positive mammography test. If a woman does not have breast cancer, the probability is 10% that she will still have a positive mammography test.

Frequency format

Ten out of every 1,000 women have breast cancer. Of these 10 women with breast cancer, 8 will have a positive mammography test. Out of the remaining 990 women without breast cancer, 99 will still have a positive mammography test

*Bayesian reasoning: vignette/case study*^39^

Typical angina chest pain: A 55year old man presented to your office with a 4 week history of sub-sternal pressure-like chest pain. The chest pain is induced by exertion, such as climbing stairs, and relieved by 3–5 minutes of rest. It sometimes radiated to the throat, left shoulder, down the arm.

1. Do you understand about the idea of sensitivity, specificity, pre-test probability, post-test probability (Yes/No)
2. What is the sensitivity of the exercise stress test?
3. What is the specificity of the exercise stress test?
4. What is the probability that this patient has significant coronary artery disease?
5. What is the probability that this patient has significant coronary artery disease if the exercise stress test is positive?
6. What is the probability that this patient has significant coronary artery disease if the exercise stress test is negative?

**Table 2:** Summary of included studies

	Total	Self-rating of understanding	Accuracy definition	Bayesian reasoning	Presentation format
Number of studies	24	2	6	22	5
Study design					
Single group	17	2	6	14	1
RCT	6	0	0	6	3
Multiple groups, unclear allocation	2	0	0	2	1
Participants					
Medical students	6	0	2	6	1
Mixed physicians	17	2	3	15	2
Single specialty	8	0	3	7	3
Other	4	0	0	4	1
How was the diagnostic information presented?					
Vignette/case study	6	0	0	6	2
Population scenario	13	0	1	12	3
Simulated patient	3	0	0	2	0
2×2 table	0	0	2	0	0
Research study extract	1	0	1	1	0
No information/unclear	3	2	2	2	0
How was understanding assessed?					
Questionnaire (multiple choice)	7	0	3	7	0
Questionnaire (open ended)	16	0	2	15	5
Interview	5	2	1	3	1
Unclear	1	0	0	1	0
Type of scenario					
Fictitious	7	0	2	7	0
Real life	16	0	2	15	5
Unclear	1	0	1	0	0
None	1	2	1	1	0
Measure of test accuracy assessed					
Sensitivity	22	2	6	20	4
Specificity/FPR	24	2	5	22	4
LR+	5	1	2	5	0
LR−	2	1	0	2	0
LR categories	1	0	0	1	0
Graphical display	2	0	0	2	1
PPV	21	1	3	19	3
NPV	6	0	1	6	1
ROC	1	1	0	0	0

FPR, false positive rate; LR−, negative likelihood ratio; LR+, positive likelihood ratio; NPV, negative predictive value; PPV, positive predictive value; RCT, randomised controlled trial; ROC, receiver operating characteristic.

### Self-reported understanding: How do doctors self-rate their understanding or use of accuracy measures?

Two studies assessed doctors self-report of their understanding or use of diagnostic information.^41^ ^45^ One study, which also contributed information on doctors’ ability to define measures of accuracy, found that 13/50 general practitioners (GPs) self-reported understanding of the definitions of sensitivity, specificity and PPV.^45^ However, when interviewed only one could define any measures of accuracy, suggesting that GPs self-rating of understanding overestimates their ability. A second study found that although 82% of doctors interviewed reported using sensitivity and specificity only 58% actually used information on sensitivity and specificity when interpreting test results and <1% reported being familiar with and using ROC curves or LRs.^41^

### Accuracy definition: “Can health professionals define measures of accuracy?”

Six single-group studies assessed health professionals’ understanding of the definition of measures of accuracy.^20^ ^21^ ^23^ ^24^ ^30^ ^45^ Four studies asked doctors to identify correct definitions of sensitivity and specificity, three using multiple choice questionnaires and one based on information provided in a research study. The proportion of doctors who correctly identified sensitivity ranged from 76% to 88%, the proportion who correctly identified specificity ranged from 80% to 88%.^20^ ^23^ ^24^ ^30^

LRs and predictive values were generally less well understood. One study comparing sensitivity, specificity and LRs found only 17% of healthcare professionals could define LR+ compared with 76% sensitivity and 80% specificity.^30^ One study found that PPV was less well understood compared with sensitivity (sensitivity 76%, PPV 61%).^20^ A study that interviewed GPs to elicit their definitions of various accuracy parameters found that only 1/13 could define PPV, 1/13 could define some aspects of sensitivity and 0/13 could define specificity.^45^ One study compared health professionals’ ability to define sensitivity, specificity, predictive values and LRs. Health professionals were less able to define predictive values and LRs compared with sensitivity and specificity.^21^ A final study, that involved asking participants to identify definitions based on a 2×2 table, reported that practicing physicians were less able to select correct definitions of sensitivity and specificity compared with medical students and research doctors but exact values were not reported.^24^

### Bayesian reasoning: “How well can health professionals combine data on pre-test probability and test accuracy to obtain information on the post-test probability of disease?”

Twenty-two studies assessed whether health professionals could combine information on prevalence with data on sensitivity and specificity (or false-positive rate) to calculate the post-test probability of disease.^17^ ^19^ ^20^ ^22–32^ ^36–42^ ^44^ Nine studies used the terms ‘sensitivity’, ‘specificity’, or ‘false-positive rate’, seven provided a text description equivalent to these terms, one used both^39^ and in five it was unclear whether terms or test descriptions were provided.^27^ ^29^ ^36–38^

Post-test estimation of probability was generally poor with a tendency to overestimation; only two studies found some evidence of successful application of Bayesian reasoning.^39^ ^40^ Thirteen studies provided data on the proportion of participants who correctly estimated the post-test probability of disease when provided with data on sensitivity and specificity (or false-positive rate) and the pretest probability of disease.^17^ ^19^ ^20^ ^23–27^ ^30^ ^32^ ^42^ ^44^ ^46^ This varied from 0% to 61%, but the proportion of study participants who did not respond was between <1% and 40%.

#### Comparison of effects of positive and negative test results on Bayesian reasoning

Fourteen studies provided test accuracy information to help with interpretation of a positive test result, one study provided information for a negative test result,^42^ and five provided information for both a positive and a negative test result.^27^ ^36^ ^37^ ^39^ ^40^ In one study it was unclear whether the test result provided should be interpreted as positive or negative^23^ and in one study participants were questioned on how they interpreted test results in general.^41^ Most participants overestimated the post-test probability of disease given a positive test result; where reported (4 studies) overestimates ranged between 46 and 73%. Two studies found that post-test probabilities were poorly estimated for positive and negative test results.^37^ ^40^ One study found that correct reasoning was applied for positive test results but that post-test probability was poorly estimated for negative test results.^39^ One study found that although the post-test probability was consistently overestimated for a positive test result, estimates were correct for negative test results.^36^ The study that assessed interpretation of a negative test result only found that 56% of participants estimated post-test probability of disease as higher than pretest probability (ie, estimate moved in the wrong direction).^42^

#### Comparison of summary metrics for Bayesian reasoning

Six studies assessed the effects of providing test accuracy information using LRs (LRs),^20^ ^27^ ^30^ ^38^ ^40^ ^44^ only two of these studies provided information on the positive LR (LR+) and the negative LR (LR−).^27^ ^40^ Three studies provided a text description rather than using the term ‘likelihood ratio’,^30^ ^40^ ^44^ and in one study a categorical approach based on the LR was used (‘quite useless’, ‘weak’, ‘good’, ‘strong’, or ‘very strong’).^38^ Two studies included an additional scenario in which the LR information was provided graphically—one provided the information as a probability modifying plot,^44^ the other as a graphic featuring five circles in a row in which an increasing number of circles were coloured black to correspond with increasing positive LRs or decreasing negative LRs.^40^

Two studies demonstrated less correct responses for post-test probability estimation with LRs (described in words in one and numerical in the other) compared with sensitivity and specificity presented numerically.^27^ ^30^ One study demonstrated similarly poor post-test probability estimation for LRs (described in words) compared with sensitivity and specificity (presented numerically).^40^ Two studies demonstrated more correct responses for post-test probability estimation with LRs (described in words or using the categorical approach) compared with sensitivity and specificity presented numerically.^20^ ^38^ ^44^ Two studies found that graphical presentation of LRs improved post-test probability estimation compared with LRs described in words or sensitivity and specificity presented numerically.^40^ ^44^

#### The effect of clinical experience, profession and academic training on Bayesian reasoning

Two studies found no effect of experience (medical students vs qualified doctors) on Bayesian reasoning,^17^ ^28^ and a further study found no influence of age.^44^ One study found that a greater proportion of newly qualified doctors were more accurate in their estimation of post-test probability (29%) compared with more experienced doctors with or without an academic affiliation (15%).^42^ Two studies demonstrated that research experience improved doctors’ ability to correctly estimate post-test probability.^24^ ^25^ One study found that midwives were less likely than obstetricians to correctly estimate post-test probability of disease.^26^

### Presentation format: “Does presenting accuracy data as frequencies and using graphic aids improve understanding compared to presenting results as probabilities?”

Five studies (3 randomised controlled trials (RCTs), 1 two-group study, and 1 single-group study) found that post-test probability estimation was more accurate when accuracy data were presented as natural frequencies^19^ ^26^ ^31^ ^32^ than as probabilities (see box 1 for example).^42^ Natural frequencies are joint frequencies of two events, for example the number of women who test positive and who have breast cancer. The same information presented as a probability would just present the probability that a woman with breast cancer has a positive test result (sensitivity), usually expressed as a percentage.^47^

Two studies^19^ ^32^ also found that health professionals spent an average of 25% more time assessing the scenarios based on a probability format compared with a natural frequency format. One RCT demonstrated that presenting test accuracy information as natural frequencies with graphical aids resulted in the highest proportion of correct post-test probability estimates (73%) compared with probabilities with graphical aids (68%), natural frequencies alone (48%) or probabilities alone (23%).^31^

## Discussion

### Statement of principal findings

This review suggests that summary test accuracy measures, including sensitivity and specificity are not well understood. Although health professionals are able to select the correct definitions of sensitivity and specificity and to a lesser extent predictive values when presented with a series of options, they are less able to verbalise the definitions themselves. LRs are least well understood, although this may reflect a lack of familiarity with these measures rather than suggesting that they are less comprehensible. Few studies found evidence of successful application of Bayesian reasoning: most studies suggested that post-test probability estimation is poor with wide variability and a tendency to overestimation for both positive and negative test results. There was some evidence that post-test probability estimation is poorer for negative than positive test results, although few studies assessed the impact of negative test results. The impact of LRs on estimation of post-test probability is unclear. Presenting data as natural frequencies rather than as probabilities improved post-test probability estimation and also the speed of calculations. The use of visual aids to present information (both on probabilities and natural frequencies) was found to further improve post-test probability estimation, although this was based on a single study. No study investigated understanding of other test accuracy metrics such as ROC curves, AUC and forest plots.

### Explanation of findings

Difficulty in interpreting summary test accuracy measures is likely to be related to their complexity. Summary test accuracy statistics used to describe test performance (eg, sensitivity and specificity and positive and negative predictive values) are conditional probabilities and misinterpretation as evidenced in this review is proposed to be a function of confusion over the subgroup of study participants the measures refer to. For example, the subgroup may be those with or without disease (sensitivity and specificity), or those with positive or with negative test results (positive and negative predictive values).

Our finding that presenting probabilities as frequencies may facilitate probability revision by healthcare professionals mirrors the findings of research carried out in the psychological literature.^18^ ^48^ ^49^ Research in the psychological literature has also shown that individuals are often conservative when asked to estimate probability revisions based on Bayes’ theorem. However, this has been shown only to be the case for information having reasonably high diagnostic value. For information with the least diagnostic value, participants are generally more extreme than would be expected based on Bayes' theorem.^50^ This is consistent with our findings where most examples presented combinations of low pretest probabilities of disease or values of sensitivity and specificity that were not sufficiently high for ruling in or ruling out disease. The findings of this review are important for those attempting to facilitate the integration of test accuracy evidence into diagnostic decision-making. Indeed qualitative research conducted recently suggests that interpretation of findings of systematic reviews of test accuracy by decision-makers is poor.^51^

### Strengths and weaknesses

To the best of our knowledge, this is the first systematic review of health professionals’ understanding of diagnostic information. We conducted extensive literature searches in an attempt to maximise retrieval of relevant studies. However, a potential limitation of our review is that the search was conducted in September 2013 and so any recently published articles will not have been captured. The possibility of publication bias remains a potential problem for all systematic reviews. Publication bias was not formally assessed in this review because there is no reliable method of assessing publication bias when studies report a variety of outcomes in different formats. However, the potential impact of publication bias is likely to be less for these types of studies where there is no clear ‘positive’ finding than for RCTs of treatment effects which may be more likely to be published if a positive association between the treatment and outcomes is demonstrated. Study quality assessment is an important component of a systematic review. For this review we did not perform a formal risk of bias assessment as study designs included in the review varied and, although we included some RCTs, most were single-group studies that examined how well doctors could perform certain calculations or understand pieces of diagnostic information. There is no accepted tool for assessing the risk of bias in these types of study and so we were unable to provide a formal assessment of risk of bias in these studies.

### Conclusions and implications for practice, policy and future research

Perhaps the more important finding of this review is the lack of understanding of test accuracy measures by health professionals. This review suggests that presenting probabilities as frequencies may improve understanding of test accuracy information and this has been embraced by both the Cochrane Collaboration^52^ and GRADE.^53^ Further research is needed to capture the needs of healthcare professionals, policymakers and guideline developers with respect to presentation of test accuracy evidence for diagnostic decision-making and how this may actually influence disease management especially as regards initiating or withholding treatment.
